# Effect of Oxidants on Properties of Electroactive Ultrathin Polyazulene Films Synthesized by Vapor Phase Polymerization at Atmospheric Pressure

**DOI:** 10.1021/acs.langmuir.2c02215

**Published:** 2022-11-29

**Authors:** Rahul Yewale, Pia Damlin, Carita Kvarnström

**Affiliations:** Turku University Centre for Materials and Surfaces (MATSURF), Department of Chemistry, University of Turku, FIN-20500 Turku, Finland

## Abstract

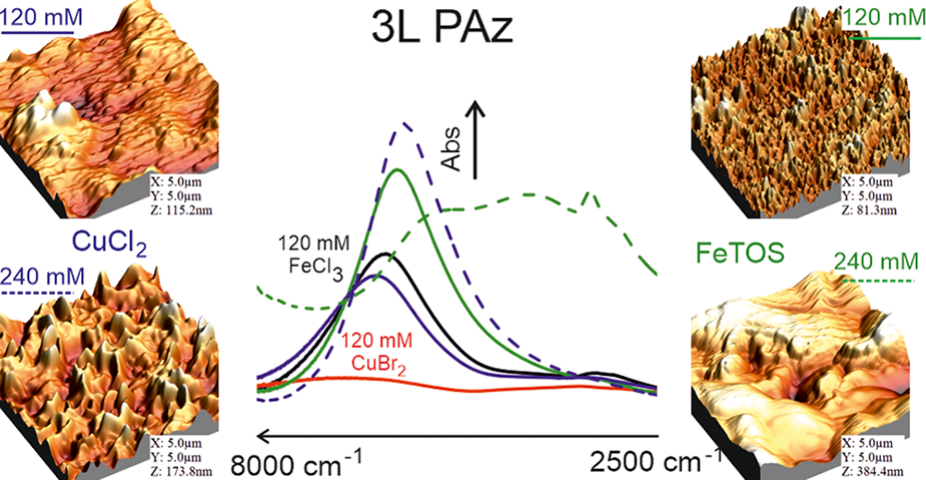

A non-benzenoid aromatic hydrocarbon azulene, naturally found in plants and mushrooms, is known for its derivatives applications in medicines. However, the processability of its chemically synthesized high-capacitance polymer is constrained by the sparingly soluble nature of its polymeric form. Oxidative chemical synthesis on a desirable substrate overcomes this difficulty. In this report, polyazulene (PAz) thin films are synthesized by vapor phase polymerization at atmospheric pressure using oxidants, such as CuCl_2_, CuBr_2_, FeCl_3_, and FeTOS. The effect of oxidants on morphologies of PAz films is studied using atomic force microscopy and microscope imaging. Each oxidant produced distinct microstructures in the films. The films synthesized using Cu(II) salts showed organized and knitted structures, whereas Fe(III) salts formed casted sheet-like disordered arrangements. The films synthesized using CuCl_2_ created uniform porous film assemblies. The pre-peak formations and their splitting observed in the cyclic voltammograms revealed phase segregations in the films. Oxidant-dependent structural and chemical differences such as charge carrier formation, doping levels, and polymer chain length in the PAz films are studied by using UV–Vis and FTIR spectroscopy. The results indicated that 240 and 180 mM are the optimum concentration of CuCl_2_ to produce high capacitance and well-organized single- and triple-layered PAz films, respectively.

## Introduction

Azulene is a non-benzenoid aromatic compound and an isomer of naphthalene. A 7-membered electron-deficient and a 5-membered electron-rich ring make azulene a polarized entity with a dipole moment of approximately 1.08 D.^1,2^ The low band gap reported in the range of 1.46 to 1.9 eV makes it an exciting material for applications in electronic devices.^3,4^ Furthermore, electrical conductivity, redox nature, and high capacitance^5,6^ (378 to 440 F/g) of polyazulene (PAz) signify its potential application in energy storage devices and as antistatic coatings. Besides, fundamental studies reported on PAz include its applications in different sensors.^7,8^ Despite the potential observed in PAz, it has not been studied as intensively as the other conducting polymers (CPs). Neoh et al. reported the first chemical synthesis of electroactive PAz using iodine and bromine.^9^ Wang et al. reported bis(cyclooctadiene)nickel(0)-catalyzed chemical synthesis of PAz by using 1,3-dibromoazulene,^10^ and Grądzka et al. synthesized PAz in various solvents using iron(III) chloride (FeCl_3_) as an oxidizing agent.^11^ So far, electrochemical^5,6,12−15^ and chemical syntheses^9−11^ of PAz and, in addition, the on-surface synthesis^16^ on gold [Au(111)] have been reported. Our recent work shows vapor phase polymerization (VPP) of PAz thin films at atmospheric pressure (AP-VPP).^17^

Techniques, such as oxidative chemical vapor deposition, VPP, Langmuir–Blodget, self-assembly, dip/spin-coating, drop casting, and on-surface and electrochemical synthesis have been used to prepare CP thin films.^18−24^ Layer-by-layer synthesis approach provides controls to engineer ultrathin films at a thickness scale of nanometers.^19,21,25^ FeCl_3_ and FeTOS are heavily used oxidants in the synthesis of CPs. In addition, iron(III)trifluoromethane sulfonate, iron(III)perchlorate, ammonium persulfate, *p*-toluenesulfonic acid, halogens, phosphomolybdic acid, ammonium cerium(IV)nitrate, cerium(IV)sulfate, 2,3-dihydroxybutanedioic acid, naphthalene sulfonic acid, and camphor sulfonic acid are oxidants that have been previously used in synthesizing CPs.

The electrochemical synthesis for PAz is well known but limited to a conducting electrode surface. The on-surface synthesis needs an ultra-high vacuum and is highly surface selective. Furthermore, the limited solubility of chemically synthesized PAz affects processability and its applications.^9^ VPP provides an alternative to overcome these obstacles for PAz thin-film synthesis.^17,22,26^ In addition, thin films of CPs synthesized using VPP acquire high transmittance, surface smoothness, and uniformness.^17,25,27^ Fabretto et al. synthesized poly(3,4-ethylene dioxythiophene) (PEDOT)-copolymer thin films of high conductivity (3400 S/cm) using vacuum VPP.^28^ In our previous work on AP-VPP PAz, the PAz films were prepared using 240 mM copper(II)chloride (CuCl_2_); the influences of various method/process parameters on film properties were studied in detail.^17^ The exciting properties, like high areal capacitance, transmittance, and low optical band gap of PAz films, synthesized using 240 mM CuCl_2_ motivated us to investigate the influence of Cu(II) and Fe(III) oxidants on the resulting PAz films.

In the present report, AP-VPP PAz films are synthesized using various concentrations of CuCl_2_, copper(II)bromide (CuBr_2_), FeCl_3_, and iron(III) *p*-toluenesulfonate (FeTOS). Additionally, the films are annealed after polymerization. A profound effect of oxidants on morphologies of the resulting PAz films is visible in atomic force microscopy (AFM) and microscope images. The fast transport of dopant ions across a film is vital for energy storage applications. Cyclic voltammograms (CVs) and relative electroactive-surface area are correlated with PAz film’s morphology associated with the oxidant type. Pre-peaks observed in the CVs of PAz are discussed with the help of the literature and experimental results. Variations in the properties of the PAz films and conformational changes are studied using Fourier-transform infrared (FTIR) spectroscopy. FTIR is an excellent basic characterization technique in ultrathin film analysis.^13,17,21^ Furthermore, the oxidation state of the films and charge carriers formed in the films are studied using UV–Vis and FTIR spectroscopy. The presented findings help to select an oxidant among Cu(II) and Fe(III) salts studied in this work for discerning applications of PAz.

## Experimental Section

### Materials

Azulene (99.7%), FeTOS hexahydrate (technical grade), FeCl_3_ (97% reagent grade), ferrocene (98%), and tetrabutylammonium tetrafluoroborate (TBABF_4_) (99%) were purchased from Sigma-Aldrich. CuCl_2_ dihydrate (99–101%) and CuBr_2_ were purchased from J. T. Baker, and CuCl_2_ dihydrate was dried in a vacuum oven. *n*-Butanol (AR grade) and pyridine (AR grade) were obtained from Lab-scan analytical sciences. Acetonitrile (MeCN) (anhydrous, 99.8%) was obtained from VWR Chemicals. Ferrocene, azulene, FeTOS, FeCl_3_, and *n*-butanol were used without further purification. TBABF_4_ was dried in a vacuum oven at 75 °C for 2 h before use. MeCN was dried using molecular sieves (4 Å, Sigma-Aldrich) for more than 24 h before electrochemical measurements.

### AP-VPP Method and Film Preparation

**Figure 1:**
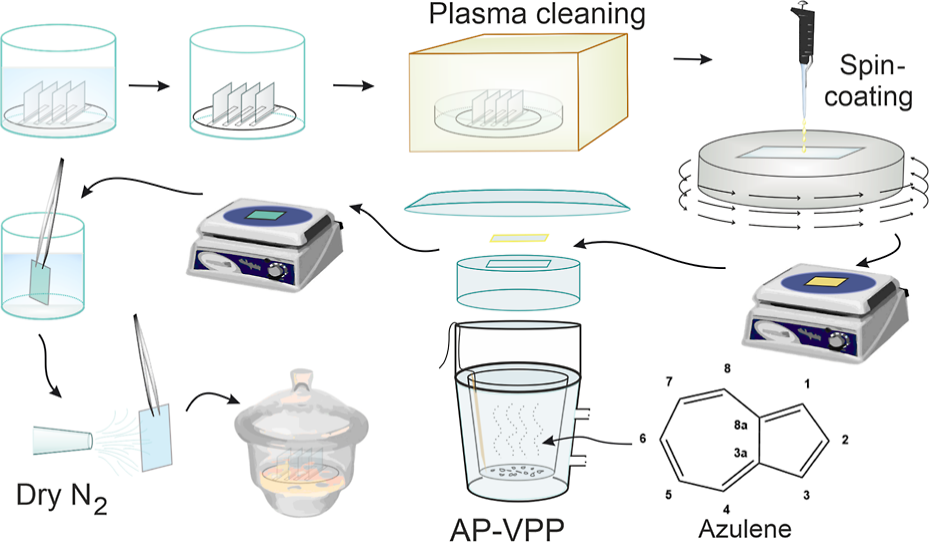
Synthesis steps in AP-VPP.

As shown in Figure 1, before the oxidant coating of the substrate, the substrates [microscope glass slides and fluorine-doped tin oxide (FTO) glass pieces] were cleaned by ultrasonication in distilled water, acetone, and ethanol, followed by drying and oxygen plasma cleaning. Next, 80 μL of oxidant solution prepared in n-butanol was dropped on a spinning substrate at 2400 rpm for 20 s and left to spin for 40 s. The oxidant-coated substrate was dried on a hot plate at 90 °C for 90 s and immediately transferred to a preheated AP-VPP cell containing azulene monomer at 57 ± 2 °C. The coated surface faced down toward the vapor. After the polymerization for 4 min, the film was annealed at 90 °C for 90 s on a hot plate in a lab atmosphere, left to cool down to room temperature, and thoroughly dip rinsed in MeCN to remove unreacted oxidants, monomers, and other byproducts of the reaction. Finally, the film was dried under a dry nitrogen gas stream. The procedure was repeated from the oxidant spin-coating step to produce multi-layered PAz films. The resulting films were always stored in a desiccator. CuCl_2_, CuBr_2_, FeCl_3_, and FeTOS were used as oxidants. The hydrates in the FeTOS salt may play the role of proton scavengers during the polymerization process.^27,29^ The concentration of 60 and 120 mM of each oxidant was used to prepare one-layered (1L) and three-layered (3L) PAz films. In addition, 1L and 3L PAz films were prepared using 180 and 240 mM of CuCl_2_ and FeTOS. We used short labeling for an easy comparison between the films. For example, the label “3L PAz (60 mM CuCl_2_)” refers to the “three-layered PAz film synthesized by using 60 mM of CuCl_2_ as an oxidant” via the synthesis mentioned above. Hereafter, all the films are explained by using their short labels. All the PAz films synthesized in this work are listed in Table S1 in the Supporting Information.

### Characterization

The UV–Vis spectra of AP-VPP PAz films prepared on glass substrates are recorded using Agilent 8453 spectrometer. The IR spectra of AP-VPP PAz films on FTO glasses were recorded by a Bruker Vertex70 FTIR spectrometer using a Harrick Seagull variable angle reflection accessory and liquid nitrogen cooled MCT (mercury-cadmium-telluride) detector at 75° angle of incidence relative to the surface normal. Spectra were recorded in the region 7500–500 cm^–1^ with 4 cm^–1^ spectral resolution. The spectrum is an average of 256 scans.

Microscope images were acquired at room temperature using a Leica microscope (20× objective). AFM measurements were carried out using a Veeco diCaliber scanning probe microscope operated in a tapping mode at room temperature. All AFM images were recorded using a Bruker TESP-MT probe (resonant freq. 320 kHz, spring const. 42 N/m, length 125 μm, width 30 μm, cantilever spec: 0.01–0.025 Ω cm antimony (n) doped silicon, 4 μm thick, and tip spec: 10–15 μm height, 8 nm radius). All AFM images of AP-VPP PAz films were analyzed in WSXM software.^30^ Cyclic voltammetry was conducted in a conventional three-electrode configuration using a one-compartment Teflon cell specially designed for FTO glass substrates in 0.1 M TBABF_4_/MeCN. PAz films on FTO glass, an Ag/AgCl wire, and a platinum wire were used as working electrodes (with an area of 1.13 cm^2^), a pseudo-reference electrode, and a counter electrode, respectively. The reference electrode was calibrated before and after every set of electrochemical measurements using the ferrocene redox couple [*E*_1/2_(Fe/Fe^+^) = 0.44 V]. CVs were recorded by a Metrohm Autolab PGSTAT 101 potentiostat at scan rates of 20, 50, 100, 150, and 200 mV/s in a potential range of −0.25 to 0.9 V against a reference electrode. All the reported CVs show the second scan of the measurements. (Notes: all solutions were purged with dry nitrogen gas for 15 min before measurement. All PAz films are handled in a lab atmosphere. Films were always stored in a desiccator after synthesis and before/after the characterization experiments.)

## Results and Discussion

### Surface Morphologies and Structural Characterization

The highly porous morphology of PAz is essential for its application as an active material in energy storage devices; it increases the active surface area and eases the transport of dopant ions across the films. Grądzka et al. synthesized PAz using FeCl_3_ in different solvents to show their influence on morphologies.^11^ Synthesis in dichloromethane resulted in porous and aggregated structures. In contrast, synthesis in water, acetonitrile, and ethanol formed compact-smooth structures, aggregates of spherical nanoparticles, and nanoparticles of 100 to 400 nm size, respectively.^11^ Suominen et al. electrosynthesized PAz in three different ionic liquids and reported two distinct porous morphologies, that is, well 3D connected small granules and poorly interconnected larger (roughly 1 μm) granules.^6^ These studies confirm that the morphology of PAz is sensitive to reaction media. The reaction medium for AP-VPP films is different from the chemically or electrochemically synthesized PAz. In AP-VPP PAz, thin-film formation occurs at the oxidant and monomer interface.^17,25,31^ In this case, the monomer and oxidant are in gaseous and solid thin-film states, respectively, providing heterogeneous reaction media.^17,25,32^ The AP-VPP cell temperature, substrate temperature, oxidant coating, washing solvents, and polymerization time play critical roles in the resulting film properties, such as surface roughness, transmittance, optical band gap, sheet resistance, and conductivity.^17,25^

**Figure 2:**
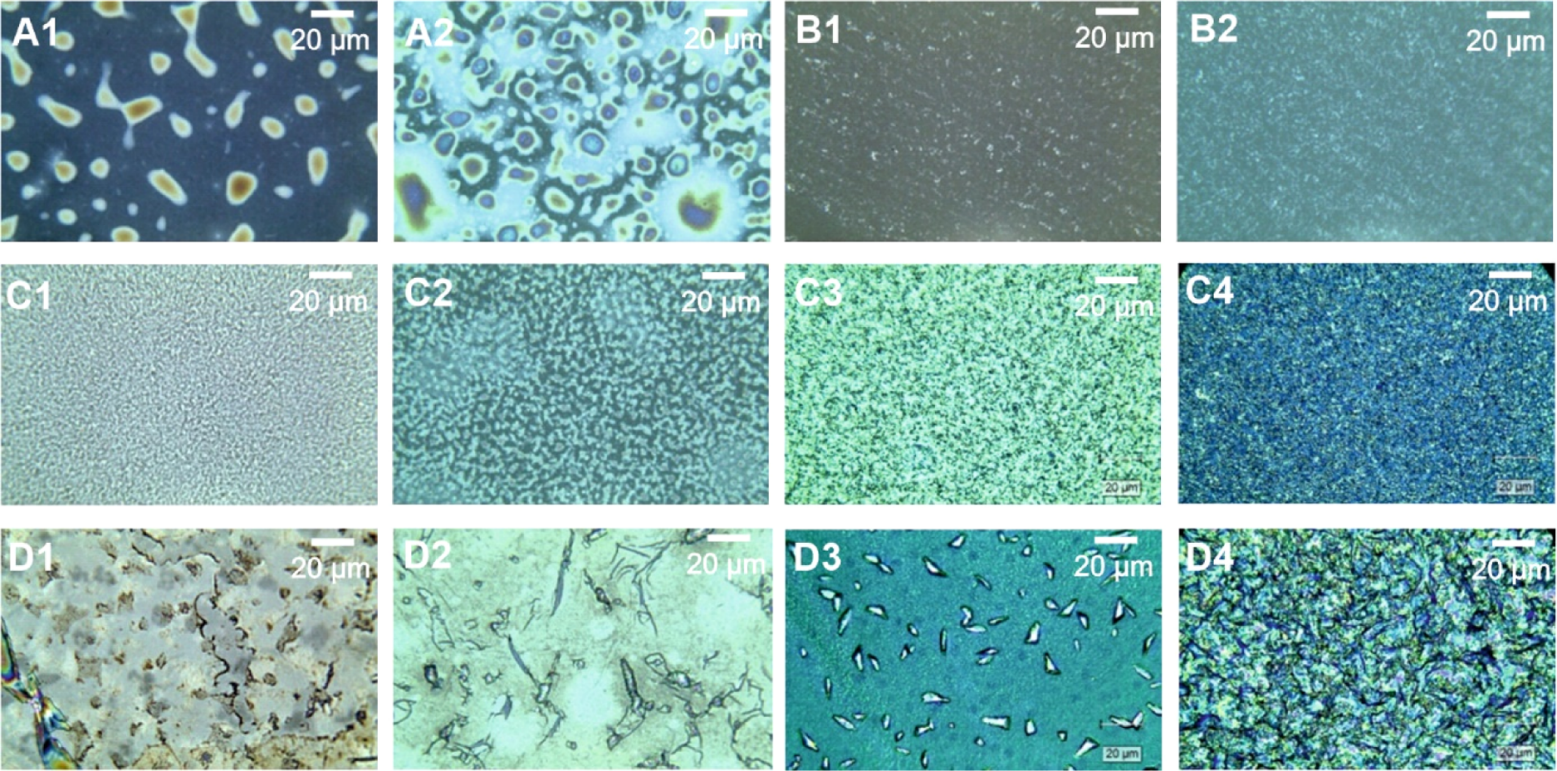
Microscopy images of 3L PAz (A1) (60 mM FeCl_3_), (A2) (120 mM FeCl_3_), (B1) (60 mM CuBr_2_), (B2) (120 mM CuBr_2_), (C1) (60 mM CuCl_2_), (C2) (120 mM CuCl_2_), (C3) (180 mM CuCl_2_), (C4) (240 mM CuCl_2_), (D1) (60 mM FeTOS), (D2) (120 mM FeTOS), (D3) (180 mM FeTOS), and (D4) (240 mM FeTOS).

**Figure 3:**
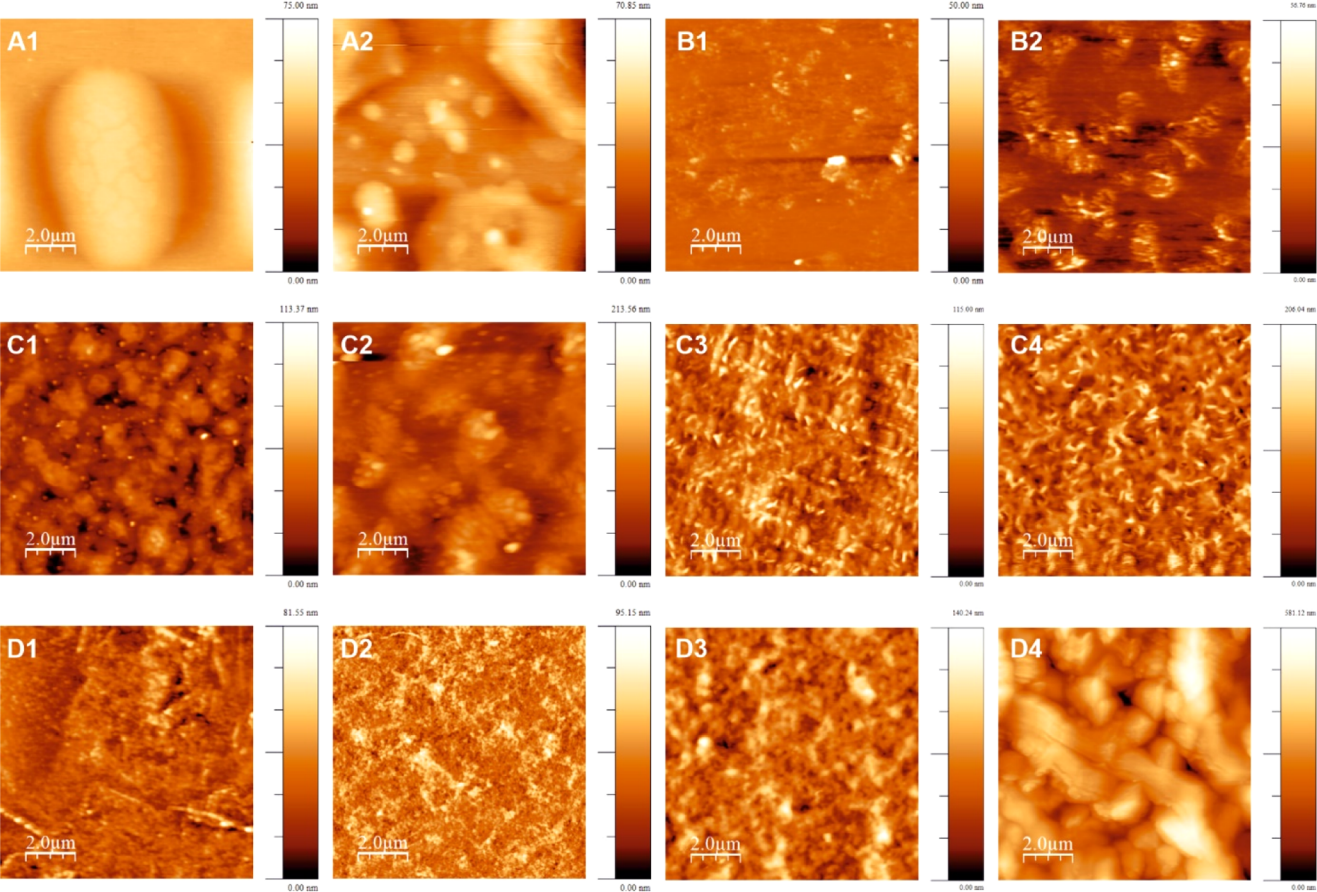
AFM images (10 × 10 μm) of 3L PAz (A1) (60 mM FeCl_3_), (A2) (120 mM FeCl_3_), (B1) (60 mM CuBr_2_), (B2) (120 mM CuBr_2_), (C1) (60 mM CuCl_2_), (C2) (120 mM CuCl_2_), (C3) (180 mM CuCl_2_), (C4) (240 mM CuCl_2_), (D1) (60 mM FeTOS), (D2) (120 mM FeTOS), (D3) (180 mM FeTOS), and (D4) (240 mM FeTOS). [50 × 50 μm AFM images are provided in Figure S2].

Microscopy images in Figure 2 show that the PAz films synthesized using FeCl_3_ formed poor-quality films (Figure 2A1). In Figure 2A2, an increase in the concentration of the FeCl_3_ oxidant accelerated the connecting PAz-island formation. The addition of layers crowded the connecting islands. A similar behavior is observed for the PAz films prepared using CuBr_2_, except that the morphologies differed entirely. CuBr_2_-synthesized films formed more scratchy and rough patches than connecting island structures (Figure 2B1,B2). In AFM images (Figure 3A1,A2), as in microscopy images, the island structure and crowding-connecting effect with size distribution due to increased oxidant concentration can be observed. A similar crowding effect accompanied by rough patches and sharp edges can be seen in the AFM images (Figure 3B1,B2) of CuBr_2_-synthesized films. The microscopy images (Figure 2C1–C4) of CuCl_2_-synthesized PAz films show more ordered film structures than FeCl_3_- and CuBr_2_-synthesized films. For PAz films synthesized using CuCl_2_, the ordered grainy structures observed in microscopy images are better resolved in AFM images (Figure 3C1–C4). The porous structure becomes more filled and organized with increasing concentrations of CuCl_2_. Figure 3C3,C4 also shows improved uniformness for 3L PAz when synthesized using 180 and 240 mM CuCl_2_.

Reduced oxyethylene ring bending vibrations upon doping with bulky tosylate ions for PEDOT and appearance of intense IR bands of out-of-plane ring deformation of =C–H at a high wavenumber for highly doped PAz indicated the steric hindrance and crowding effect of bulky dopant ions in the respective polymer films.^17,25^ In the present work, partially doped PAz films with bulky tosylate anions synthesized using FeTOS also show induced stress in the film structures. This stress-induced strain causes ruptures during the film-washing step even though the films are annealed. As a result, uniform sheet-like broken and overlapped structures can be observed in both microscopy (Figure 2D1–D4) and AFM images (Figure 3D1–D4) of FeTOS-synthesized PAz films. These images show that the thin uniform sheets of PAz become complex and globular in the structure with the increasing concentration of FeTOS due to overlapping broken structures. After the washing step, the overlapping and stacking of broken structures must have promoted further disorder with multiple layers of PAz films. The CuCl_2_- and FeTOS-synthesized 1L PAz films (Figure S1) show similar morphological trends as their respective 3L PAz films, but the trends are more pronounced in 3L PAz films and with the growing oxidant concentration. The multiplying effect of added layers must justify pronounced trends in 3L PAz films.

All four oxidants used in the present work developed distinct morphologies in 1L and 3L PAz films. Here, they are differentiated and grouped as films synthesized using Cu(II) and Fe(III) oxidants. The comparison between these two suggests that Cu(II) forms films of more organized and knitted structures, whereas Fe(III) forms casted sheet-like disordered structures. A standard electrode potential for Cu^2+^/Cu^+^ is 0.159 V, whereas for Fe^3+^/Fe^2+^, it is 0.771 V.^33^ A higher reduction potential of Fe(III) indicates that the Fe(III) oxidants are stronger than the Cu(II) oxidants. Consequently, well-organized structures of Cu(II)-synthesized PAz films could be an attribute of slow polymerization. Another aspect of FeCl_3_ and CuCl_2_ is that they supply the film with a common dopant ion Cl^–^, whereas CuBr_2_ and FeTOS supply Br^–^ and TOS^–^ ions for doping, respectively. The use of Cl^–^ with Fe(III) caused small sheet-like islands of PAz, whereas bulky TOS^–^ formed big casted sheets in the films. Br^–^ with Cu(II) formed isolated grainy patches of PAz, whereas Cl^–^ formed ordered, well-connected repeating units of porous structures in the PAz films. The notable difference between Cl^–^, Br^–^, and TOS^–^ is the size of the anion. TOS^–^ is much bulkier than Cl^–^ and Br^–^. The topological polar surface area computed by Cactvs 3.4.8.18 for TOS^–^ is 65.6 Å^2^, whereas for Cl^–^ and Br^–^ it is 0 Å^2^.^34,35^ The differences in nano- to microstructural arrangements of PAz films also reveal that their electrochemical properties may differ.

### Electrochemical Characterization by CV

**Figure 4:**
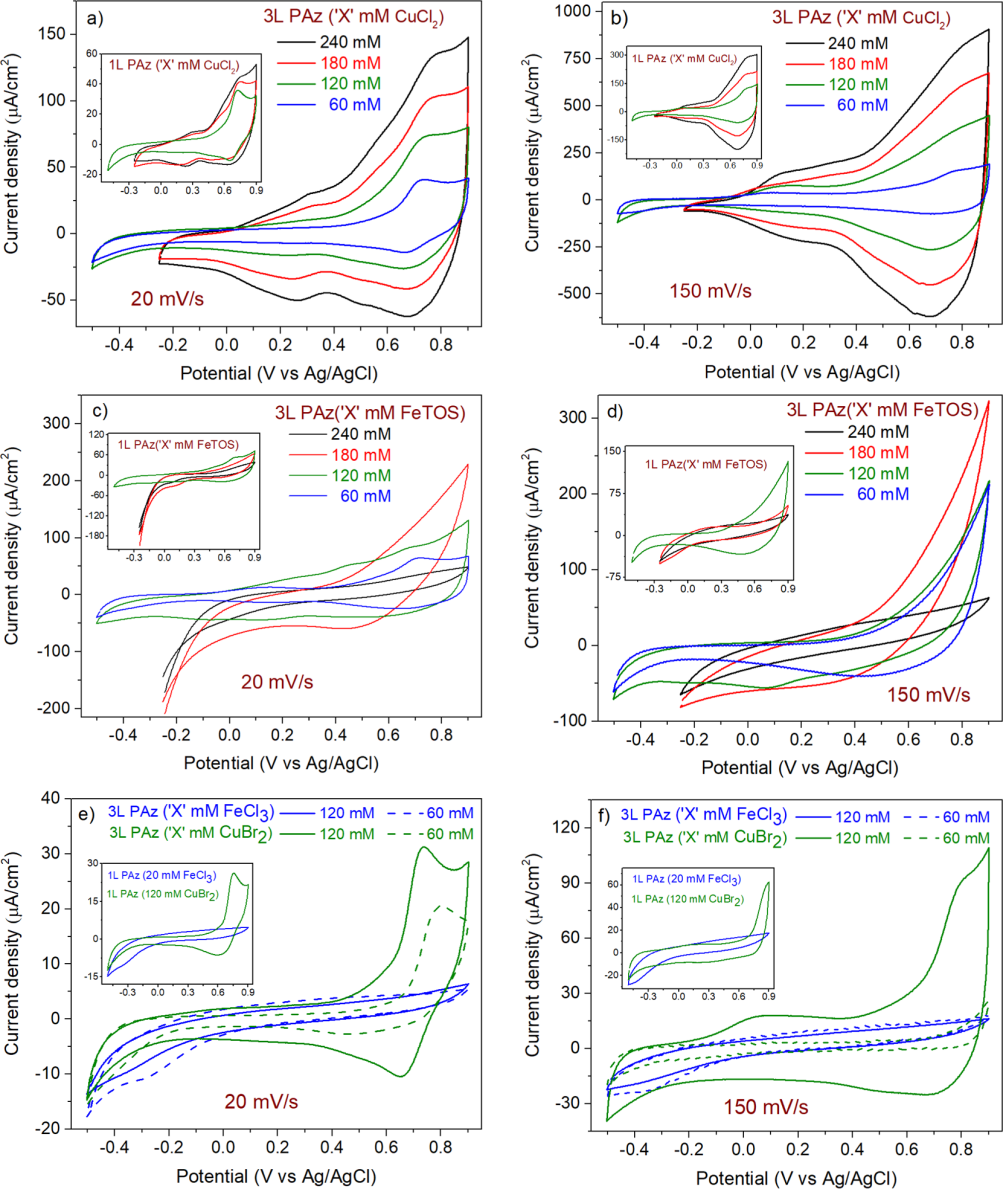
CVs of 3L PAz (60, 120, 180, and 240 mM CuCl_2_) films are shown at scan rates of (a) 20 and (b) 150 mV/s. CVs of 3L PAz (60, 120, 180, and 240 mM FeTOS) films are shown at scan rates of (c) 20 and (d) 150 mV/s. CVs of 3L PAz (60 and 120 mM FeCl_3_) and 3L PAz (60 and 120 mM CuBr_2_) films are shown at scan rates of (d) 20 and (e) 150 mV/s. The insets show CVs of 1L PAz films with the same axis labels and line colors as their corresponding 3L PAz films.

Figure 4a,b shows that the current densities in the CVs of 1L and 3L PAz films increased as the concentration of CuCl_2_ used in the film synthesis increased. It shows the increased deposition of electroactive PAz with increasing order of CuCl_2_ concentration in the synthesis. In addition, growth in current densities due to the increasing scan rate and fast and reversible redox processes during the p-doping can be observed in the CVs of PAz films synthesized using CuCl_2_ (Figures 4a,b and S3A3,B3,C3,D1,D3,E1,E3). In contrast, Figure 4b,c shows that the current densities in the CVs of 1L and 3L PAz films deteriorated as the concentration of FeTOS used in the synthesis increased, indicating inadequate growth of electroactive PAz. Besides, compared to PAz films synthesized using CuCl_2_, the insignificant growth in current densities at high scan rates and the p-doping for PAz films synthesized using FeTOS show slow redox processes (Figures 4b,c and SI3A4,B4,C4,D2,D4,E2,E4). In Table 1, the lowest drop (10 to 45%) in areal capacitance (*C*_A_) at an elevated scan rate was observed for PAz films synthesized using CuCl_2_. In contrast, the highest drop (77 to 94%) was observed for films synthesized using FeTOS. This drop in *C*_A_ at the elevated scan rate was diminished for the PAz films synthesized using high concentrations of CuCl_2_. Furthermore, the electroactivity of PAz films synthesized using 60 and 120 mM FeTOS was better compared to films synthesized using 180 and 240 mM FeTOS. In Figure 4c,d, 3L PAz (120 mM CuBr_2_) shows similarities in electroactivity with films synthesized using CuCl_2_ except for the low capacitance (Figure S3C2). The poor quality of film deposition seen in the morphology analysis of PAz films synthesized using FeCl_3_, 3L PAz (60 mM CuBr_2_), and 1L PAz (120 mM CuBr_2_) reflected in their respective CVs showing insignificant increase in current densities and poor doping–dedoping processes (Figure S3A1,A2,B1,B2,C1). Among these films, the highest *C*_A_ of 3.2 and 4.52 mF/cm^2^ were observed for 3L PAz (180 and 240 mM CuCl_2_) and 4.06 mF/cm^2^ for 3L PAz (120 mM FeTOS) (Table 1). These values are comparable with previously reported 3.75 mF/cm^2^ for 3L PAz and 5.2 mF/cm^2^ for 3L PEDOT films synthesized using AP-VPP.^17,25^ The electrochemically synthesized thicker PAz films exhibited areal capacitance 3 to 10 times higher than the AP-VPP PAz films (27 to 147 nm in thickness).^6,17^ However, a volumetric capacitance of 704 ± 42 F/cm^3^ of AP-VPP PAz films justifies its potential for energy storage devices.^17^

**Table 1:** List of Charge and Areal Capacitance (*C*_A_) Values Calculated from the CVs of PAz Films Synthesized Using Various Oxidants at Low (20 mV/s) and High (200 or 150 mV/s) Scan Rates

Scan rate mV/s	AP-VPP PAz layers	Oxidant used in synthesis	Charge (mC)	Areal capacitance (*C*_A_ in mF/cm^2^)	% Drop in *C*_A_ at high scan rate compared to the values obtained at low scan rate
20	1L	240 mM CuCl_2_	1.82	1.40	
200			1.64	1.26	10
20	3L		5.88	4.52	
200			5.05	3.89	14
20	1L	240 mM FeTOS	2.92	2.25	
200			0.25	0.19	92
20	3L		3.75	2.89	
200			0.37	0.29	90
20	1L	180 mM CuCl_2_	1.66	1.28	
200			1.1	0.85	34
20	3L		4.16	3.20	
200			3.69	2.84	11
20	1L	180 mM FeTOS	4.07	3.13	
200			0.24	0.18	94
20	3L		9.08	6.99	
200			0.91	0.70	90
20	1L	120 mM CuCl_2_	1.09	0.84	
200			0.60	0.46	45
20	3L		2.69	2.07	
200			2.17	1.67	19
20	1L	120 mM FeTOS	2.57	1.98	
200			0.34	0.26	87
20	3L		5.28	4.06	
200			0.45	0.34	92
20	1L	120 mM CuBr_2_	0.62	0.48	
200			0.15	0.12	76
20	3L		0.91	0.70	
200			0.41	0.31	55
20	1L	120 mM FeCl_3_	0.29	0.22	
200			0.12	0.09	60
20	3L		0.30	0.23	
200			0.10	0.08	65
20	3L	60 mM CuCl_2_	1.33	1.02	
150			0.90	0.69	33
20	3L	60 mM FeTOS	2.56	1.97	
150			0.59	0.45	77
20	3L	60 mM CuBr_2_	0.43	0.33	
150			0.06	0.05	85
20	3L	60 mM FeCl_3_	0.36	0.28	
150			0.13	0.10	63

**Figure 5:**
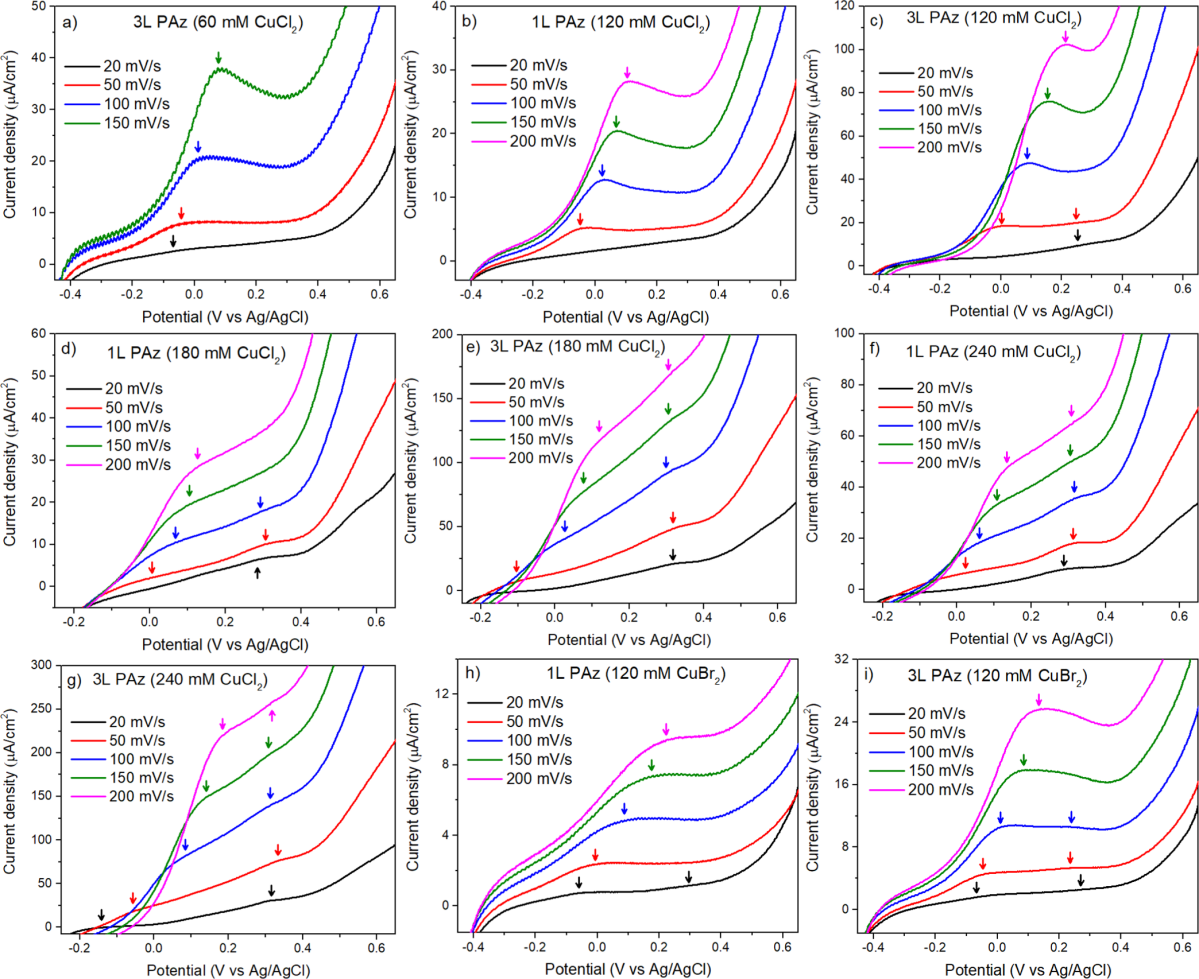
(a) 3L PAz (60 mM CuCl_2_), (b) 1L PAz (120 mM CuCl_2_), (c) 3L PAz (120 mM CuCl_2_), (d) 1L PAz (180 mM CuCl_2_), (e) 3L PAz (180 mM CuCl_2_), (f) 1L PAz (240 mM CuCl_2_), (g) 3L PAz (240 mM CuCl_2_), (h) 1L PAz (120 mM CuBr_2_), and (i) 3L PAz (120 mM CuBr_2_) showing the shape of pre-peak formations in the CV during the forward scan.

The electrochemical p-doping of electroactive thin films deposited on the electrode surface (FTO glass) shows pre-peaks (Figure 5) followed by the main oxidation peak (Figure S3). Various justifications, such as adsorption phenomena, relaxation, phase variation, step-reduction, charge trapping, and different chain lengths, have been suggested for pre-peak formation in CVs of CPs.^33,36−41^

In Figure 5, the pre-peaks suggest multiple phases of PAz existing in the films, which could be a function of disorder in structural arrangements or the existence of segments with different conjugations or contributions by both. Lete et al. reported different morphologies for electrosynthesized PAz films depending on switching potentials. A low switching potential (1.2 V) resulted in a more homogeneous film surface than the high switching potential (1.8 V).^42^ Son et al. reported that the electrochemically induced slower deposition of poly(3-hexylthiophene) led to lateral thin-film formation on energetically preferred positions of the electrode. However, the rapid oxidation rate produced vertically preferred three-dimensional polymer growth.^38^ The same work showed that the pre-peak is from the laterally adsorbed redox phases, whereas the main peak is a result of the vertically grown phases.^38^ Skompska et al. synthesized poly(3-methyl thiophene) films using different charge densities. Thick films showed one main peak due to the oxidation of short polymer chains and a second broad peak due to the wide distribution of redox energies of different conjugation lengths.^36^ The disordered parts of the thick films generated porous structures, which were more accessible for ion penetration than the thin films.^36^ Österholm et al. found that higher viscosity of the room-temperature ionic liquids slowed down the PAz film deposition and formed a smoother high-quality polymer film with segments of longer conjugation length.^43^ These studies suggest that the slow polymerization rate led to consistent homogeneous horizontal growth of films with an extended conjugation, forming a pre-peak in the CVs. In contrast, fast deposition led to inhomogeneous, porous, and vertical growth of short-chain polymers, contributing to the main oxidation peak. This explanation is analogous to pre-peak, explained by the kinetic charge trapping concept. The confinement of charges explains charge trapping in the polymer film out of defects, where the polymers of different redox potentials other than the main polymer are formed in the film, or the outer layer does not undergo redox processes before the inner layer causing the development of potential gradient across the film.^39−41^

Figure 5 shows that for AP-VPP PAz films, an increase in the scan rate causes a shift in the pre-peak toward higher potential. The 3L PAz (60 mM CuCl_2_) and 1L PAz (120 mM CuCl_2_) showed a single pre-peak formation (Figure 5a,b). The 3L PAz (120 mM CuCl_2_) showed slight peak splitting at 50 mV/s, whereas it showed a single pre-peak at higher scan rates (Figure 5c). In 1L PAz (180 mM CuCl_2_), splitting is observed for 50 and 100 mV/s, whereas in 3L PAz (180 mM CuCl_2_), it can be observed at all scan rates (Figure 5d,e). The splitting is visible at all scan rates in CVs of 1L and 3L PAz (240 mM CuCl_2_) (Figure 5f,g). In all these films, peak splitting was dominant at low scan rates. In 1L PAz (120 mM CuBr_2_), it is slightly visible at 20 mV/s, whereas in 3L PAz (120 mM CuBr_2_), it is visible until 150 mV/s (Figure 5h,i). These observations suggest that the splitting is dominant at low scan rates in 3L films synthesized using high oxidant concentrations. Higher oxidant concentrations and multiple layers probably form thicker films and phase separation. These phases get enough time to show individual appearances in the CVs at a slow scan rate. A single broad peak is formed at a high scan rate if the material is not conducting enough. No pre-peak was observed in the CVs of PAz films synthesized using FeCl_3_ (Figure S3). In 3L PAz (60 mM FeTOS) and 1L PAz (120 mM FeTOS), a single pre-peak is observed at 20 and 50 mV/s scan rate and in 3L PAz (120 mM FeTOS), splitting is observed at 20 mV/s (Figure S3A4–C4). CVs for 1L and 3L (180 and 240 mM FeTOS) did not show current peaks (Figure S3D4,E4). The limited reversibility of the material is due to slow and constrained ion penetration and conformational changes upon doping, which can be justified by compact sheet-like structures observed in morphological studies. As a result, PAz films synthesized using a low FeTOS concentration show peaks only at low scan rates. Thick 1L and 3L films synthesized using high-concentration FeTOS show no peaks and poor electroactivity. Spectroscopic methods effectively probe the optical and electronic properties and the structural changes in CP films. The following sections discuss the UV–Vis and FTIR spectra of 1L and 3L PAz (180 and 240 mM FeTOS). They also showed significant differences when comparing 1L and 3L PAz (60 and 120 mM FeTOS).

### UV–Vis Spectra of PAz Films

**Figure 6:**
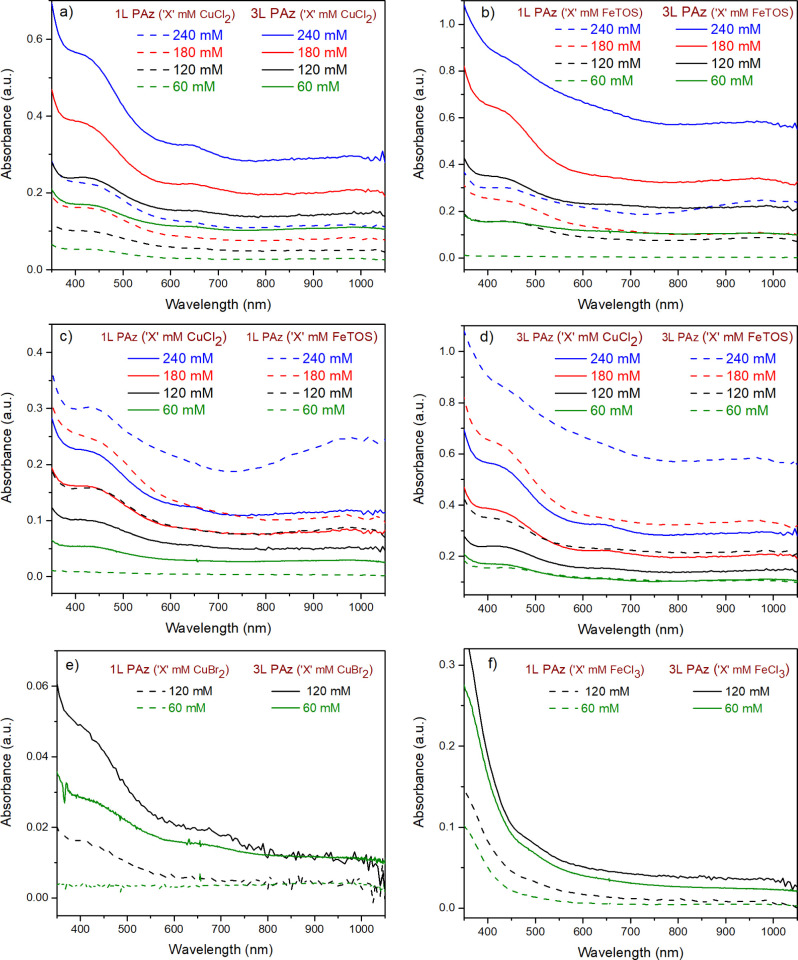
UV–Vis spectra of (a) 1L PAz (dashed lines) and 3L PAz (solid lines) (60, 120, 180, and 240 mM CuCl_2_), (b) 1L PAz (dashed lines) and 3L PAz (solid lines) (60, 120, 180, and 240 mM FeTOS), comparison of UV–Vis spectra of 1L PAz (60, 120, 180, and 240 mM CuCl_2_) vs 1L PAz (60, 120, 180, and 240 mM FeTOS) and a similar comparison for 3L PAz films are shown (c,d), respectively. UV–Vis spectra of (e) 1L PAz (dashed lines) and 3L PAz (solid lines) (60 and 120 mM CuBr_2_) and (f) 1L PAz (dashed lines) and 3L PAz (solid lines) (60 and 120 mM FeCl_3_).

The neutral part of PAz shows absorbance around 450 nm.^44−46^ In this region, the growing absorbance of 1L and 3L PAz films with increased synthesis concentration from 60 to 240 mM of CuCl_2_ and FeTOS can be observed in Figure 6a,b. It suggests the enhanced deposition of PAz with increasing concentrations of CuCl_2_ and FeTOS. In Figure 6 e,f, the increase in absorbance around 450 nm with the concentrations of FeCl_3_ and CuBr_2_ is not as significant as for the other two oxidants. The absorbance from a neutral part of PAz synthesized using CuBr_2_ is very low, indicating poor film deposition. The absorbance around 640 nm and above is due to the charge carriers’ formation in the film upon doping.^44−47^Figure 6 shows differences in the absorbance at 640 nm and above for PAz films synthesized using the four different oxidants. This indicates that all four oxidants produced different doping states in the resulting PAz films. The comparison between the absorbance around 450 and 640 nm shows that PAz films synthesized using 60 and 120 mM CuBr_2_ are less doped than those synthesized using 60 and 120 mM of FeCl_3_ (Figure 6e,f). A similar comparison in Figure 6a, for 1L and 3L PAz films synthesized using 60 to 240 mM, CuCl_2_ shows that the neutral form of the film dominates over the doped form with increasing CuCl_2_ concentration. In PAz films synthesized using FeTOS (Figure 6b), 1L and 3L (240 mM FeTOS) displayed drastic increments in absorbance. The continuous absorbance observed for 3L PAz (240 mM FeTOS) reveals the highly doped state of the film. 1L PAz (240 mM FeTOS) shows absorbance above 750 nm as significant as in its neutral part and in 3L PAz (240 mM FeTOS), the neutral part is slightly dominating. 1L and 3L (60, 120, and 180 mM FeTOS) show consistent growth in the absorbance range except for the 3L (180 mM FeTOS), which shows significant absorbance around 450 nm due to the neutral part of PAz. Additionally, in Figure 6c,d, a comparison of absorbance of the neutral part and above 640 nm in PAz films synthesized using CuCl_2_ and FeTOS suggests that FeTOS produces highly oxidized PAz films with high absorbance compared to the films deposited by using CuCl_2_ as an oxidant.

### FTIR Spectra of PAz Films

Infrared spectroscopy offers insights into doping-induced structural changes because of strong electron–phonon coupling and doping-induced symmetry breaking in conjugated polymers.^13,14,48−50^ Doping of conjugated polymers leads to charge carrier formation in the polymer resulting in a polarization of the chains that introduces new doping-induced infrared active vibration (IRAV) bands between 2000 and 200 cm^–1^. Additionally, electronic mid-gap transitions give rise to electronic absorption bands in the spectral range above 2000 cm^–1^.^47,51−54^ The delocalization of doping-induced charge carriers and the IRAV bands are associated with the effective conjugation length.^13,14,47−50^

### Absorption in the NIR Region

Nöll et al. reported the shift from 7000 to 4700 cm^–1^ and from 6250 to 6500 cm^–1^ in absorption maxima of radical cations and dications of azulene oligomers, respectively, with an increase in the chain length.^45^ It shows that with the increase in chain length, the absorbance due to the radical cations tends to shift significantly to lower wavenumbers. In contrast, absorbance due to dications has a propensity to shift slightly to a higher wavenumber. In the same studies, chemically synthesized PAz showed absorbance at 4300 and 6000 cm^–1^ at low and medium doping levels (electrochemical potential), respectively.^45^ It can be interpreted as the formation of radical cations dominating at low doping and radical dications at medium doping.

The in situ FTIR-ATR difference spectra obtained upon p-doping of electro-synthesized PAz at −0.6 to 1.2 V by Meana-Esteban et al. showed that the broad absorbance band around 7000 cm^–1^ continuously increased with applied potential and shifted toward low energy forming two slightly distinct absorption maxima at 3700 and at 6200 cm^–1^ at the high electrochemical potential.^13^ In this case, the 3700 cm^–1^ band must be arising due to the radical cations and 6200 cm^–1^ due to the radical dications. In the same studies, the PAz film synthesized at a higher potential (−0.6 to 1.8 V) with higher cross-linking and extended effective conjugation length showed two electronic absorption maxima during the p-doping of the PAz film. The band at 5600 cm^–1^ shifted to a lower wavenumber with increased applied potential. The absorbance maxima at around 3800 cm^–1^ dominated until 0.5 V, and 5200 cm^–1^ dominated the spectrum after 0.6 V.^13^ Similarly, the band at 3800 and 5200 cm^–1^ must originate from the absorption by radical cations and dications, respectively. In spite of cross-linking-driven extended effective conjugation length with an increase in electrochemical synthesis potential, the shifts in radical cation absorbance to a higher wavenumber and radical dication absorbance to a lower wavenumber suggest the reduced polymer chain length in PAz films. This observation could be explained by the degradation phenomena at the high doping level of cross-linked PAz films.^13,45^

### PAz Films Synthesized Using FeTOS

**Figure 7:**
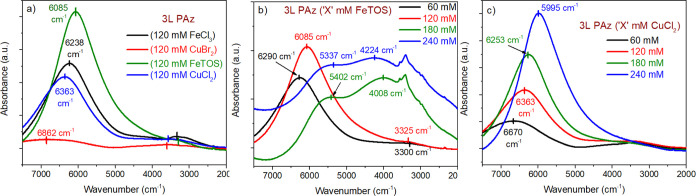
FTIR spectra of (a) 3L PAz (120 mM FeCl_3_, CuBr_2_, FeTOS, and CuCl_2_), (b) 3L PAz (60, 120, 180, and 240 mM FeTOS), and (c) 3L PAz (60, 120, 180, and 240 mM CuCl_2_) in the range 7500 to 2000 cm^–1^.

In Figure 7b, the absorption maxima at 6290 cm^–1^ in 3L PAz (60 mM FeTOS) shifts to 6085 cm^–1^ in 3L PAz (120 mM FeTOS), and it further shifts to 5402 cm^–1^ in 3L PAz (180 mM FeTOS) and to 5337 cm^–1^ in 3L PAz (240 mM FeTOS). The absorption band between 2600 and 4000 cm^–1^ (centered around 3300 cm^–1^) in 3L PAz (60 mM FeTOS) and (120 mM FeTOS) shifts and grows in intensity to 4008 cm^–1^ in 3L PAz (180 mM FeTOS) and to 4224 cm^–1^ in 3L PAz (240 mM FeTOS). The shifts in radical dications absorbance from 6290 to 5337 cm^–1^ and radical cations absorbance from 3300 to 4224 cm^–1^ with increasing concentration of FeTOS used in synthesis indicate a trend of reduced polymer chain length in resulting PAz films. The shift is significant for 180 and 240 mM FeTOS compared to 60 to 120 mM FeTOS, indicating some drastic change in PAz films. This change can be noticed in morphologies, UV–Vis spectra, and CVs of these films, as 3L PAz (180 and 240 mM FeTOS) showed poor electroactivity. The spectra of 60 and 120 mM FeTOS-synthesized 3L PAz films are dominated by radical dications, whereas the spectra of 180 and 240 mM FeTOS-synthesized 3L PAz films are dominated by both radical cation and dication formations. It suggests the medium doping state for 3L (60 and 120 mM FeTOS), whereas the low to medium doping state for 3L (180 and 240 mM FeTOS). All these observations can be explained by considering the transformations in the polymer as a result of high oxidant concentration-induced fast polymerization and high oxidized form of the resulting PAz films, leading to irreversible changes.^13,17,45^ The decomposition of PAz at a high doping level could be due to the additional cross-linking in polymer chains.^13,45^ Therefore, despite the highly oxidized form of 3L (180 and 240 mM FeTOS) films, they showed absorbance in both low and medium doping forms.

### PAz Films Synthesized Using CuCl_2_

In Figure 7c, CuCl_2_-synthesized PAz films show two absorption bands between 7000 and 2600 cm^–1^. The absorption maxima at 6670 cm^–1^ in 3L PAz (60 mM CuCl_2_) shifts to 6363 cm^–1^ in 3L PAz (120 mM CuCl_2_), to 6253 cm^–1^ in 3L PAz (180 mM CuCl_2_), and to 5995 cm^–1^ in 3L PAz (240 mM CuCl_2_). The weak absorption band between 2800 and 4000 cm^–1^ does not show a significant variation with an oxidant concentration. As described in the previous section, the spectra of CuCl_2_-synthesized films are dominated by radical dications, suggesting that all the films are at a medium doping state. The shift from 6670 to 5995 cm^–1^ with increasing concentrations of CuCl_2_ in synthesis is analogous to the increase in electrochemical synthesis potential. It indicates reduced polymer chain length in PAz films even if there is extended effective conjugation with increased CuCl_2_ concentration during the synthesis.

### Comparison between All the Oxidants and IRAV Bands

The broad absorption maxima at 6862 cm^–1^ in 3L PAz (120 mM CuBr_2_) in Figure 7a, appears at 6363 cm^–1^ in 3L PAz (120 mM CuCl_2_), 6238 cm^–1^ in 3L PAz (120 mM FeCl_3_), and 6085 cm^–1^ in 3L PAz (120 mM FeTOS) indicating the order of doping level^13,45^ is highest in 3L PAz (120 mM FeTOS) followed by 3L PAz (120 mM FeCl_3_) > 3L PAz (120 mM CuCl_2_) and lowest in 3L PAz (120 mM CuBr_2_). In Figure 7a, the weak absorption band between 5000 and 2800 cm^–1^ is much broader and distinctive for 3L PAz (120 mM CuBr_2_) than for the other films. This band appears at a lower wavenumber in 3L PAz (120 mM FeCl_3_) and (120 mM FeTOS). It suggests the extension in conjugation length from 3L PAz (120 mM CuBr_2_) and (120 mM CuCl_2_) to 3L PAz (120 mM FeCl_3_) and (120 mM FeTOS).^45^

**Figure 8:**
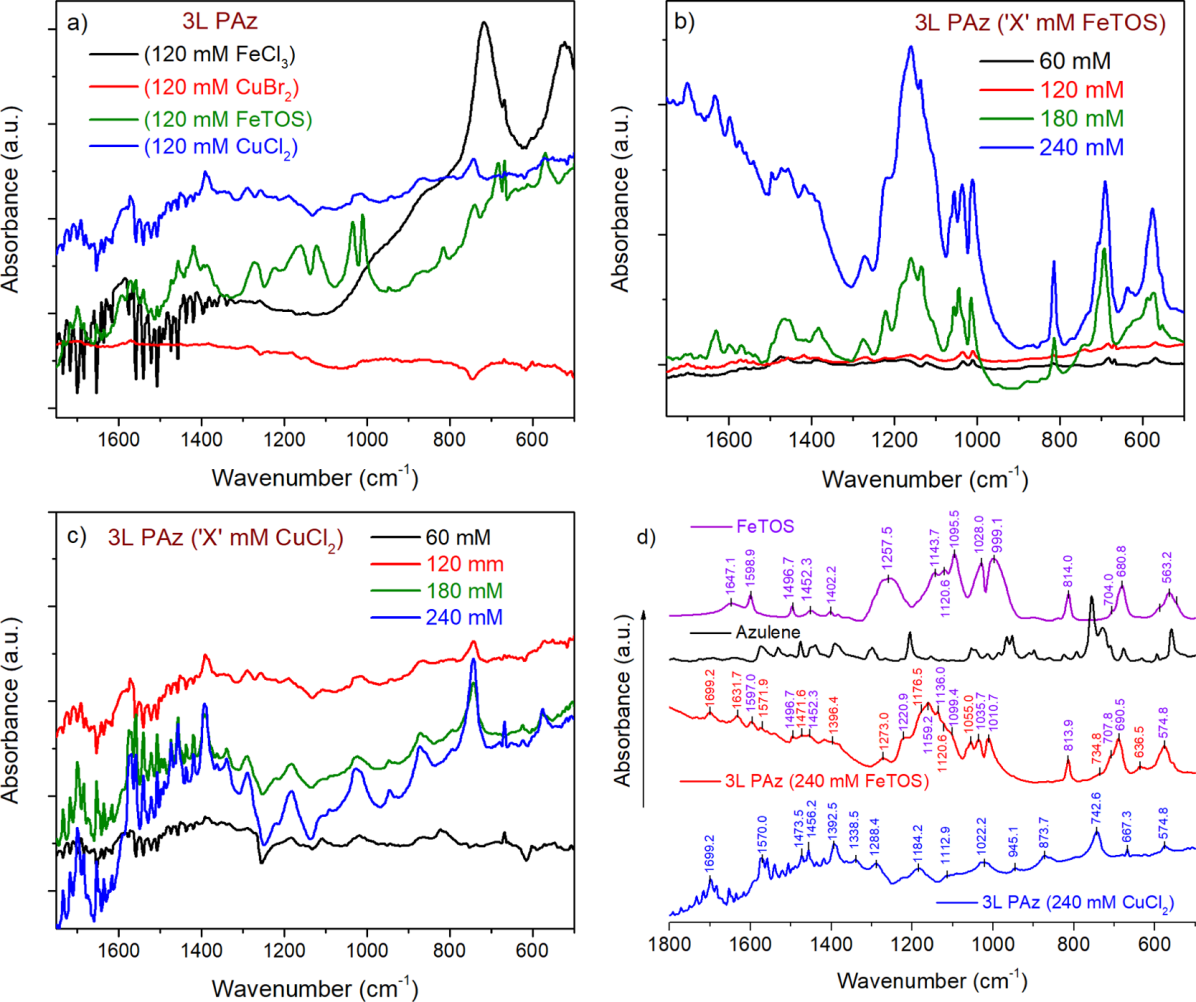
FTIR spectra of (a) 3L PAz (120 mM FeCl_3_, CuBr_2_, FeTOS, and CuCl_2_), (b) 3L PAz (60, 120, 180, and 240 mM FeTOS), and (c) 3L PAz (60, 120, 180, and 240 mM CuCl_2_) in the range 1750 to 500 cm^–1^. Comparison of FTIR spectra of (d) 3L PAz (240 mM FeTOS and CuCl_2_), azulene, and FeTOS, in the range 1800 to 500 cm^–1^.

In Figure 8a, an increase in the intensity of IRAV bands^17^ is visible in 3L PAz (120 mM FeTOS) and slightly in 3L PAz (120 mM CuCl_2_). These bands are not recognizable in 3L PAz (120 mM FeCl_3_) and 3L PAz CuBr_2_ films, which could be due to the poor film formation and PAz in the less doped form. The IRAV bands observed for 3L PAz (240 mM FeTOS and CuCl_2_) are marked in Figure 8d. In Figure 8b, the intensity of IRAV bands grows insignificantly in 3L PAz (120 mM FeTOS) compared to 3L PAz (60 mM FeTOS). In contrast, these bands become significantly intense in 3L PAz (180 mM FeTOS) and (240 mM FeTOS). Similar drastic behavior at these FeTOS oxidant concentrations is also observed in morphologies, CVs, UV–Vis, and NIR spectra. Intense IRAV bands of 3L PAz (180 mM FeTOS) and (240 mM FeTOS) confirm the polymer chain polarization due to charge carriers’ formation in the polymer upon high doping levels in the films. In Figure 8c, the IRAV bands for 3L PAz synthesized using 60 to 240 mM CuCl_2_ show consistent development in intensity and structure. It indicates linear growth in the doping forms of these films. The same linearity in the properties of PAz films synthesized using CuCl_2_ is also observed in morphologies, CVs, UV–Vis, and NIR spectra.

Figure 8d compares IRAV bands observed for 3L PAz (240 mM FeTOS and CuCl_2_) with the spectra for monomer azulene and oxidant FeTOS. The sharp peaks in the IR spectrum of azulene show peak broadening in the polymerized form. The IR spectrum of the PAz film synthesized using FeTOS shows additional peaks (marked in purple) originating from TOS^–^ dopant anions, whereas such peaks are not visible in the spectrum of the CuCl_2_-synthesized Cl^–^ doped-PAz film. The in-plane ring deformations at 1022, 1113, 1184, and 1288 cm^–1^ due to =C–H, and stretching vibrations at 1338, 1393, 1456, 1474, and 1570 cm^–1^ due to C=C in 3L PAz (240 mM CuCl_2_) show a blue shift in 3L PAz (240 mM FeTOS). These blue shifts and very weak bands of out-of-plane ring deformation due to =C–H in high-concentration FeTOS-synthesized PAz films also confirm overoxidation and poor electroactivity of the film. The out-of-plane ring deformations distinctly appear in PAz films synthesized using CuCl_2_ (743, 874, and 945 cm^–1^).

## Conclusions

Electroactive thin films of PAz are synthesized by AP-VPP using CuCl_2_, CuBr_2_, FeCl_3_, and FeTOS as oxidants. A comparison of PAz film morphologies synthesized using Cu(II) and Fe(III) oxidants showed that the slow polymerization accomplished by using Cu(II) oxidants formed films with well-organized and knitted microstructures. In contrast, fast polymerization by Fe(III) oxidants produced compact and disordered islands with broken and overlapped sheet-like structures. In addition, dopant ions (Cl^–^, Br^–^, and TOS^–^) incorporated in the films during the synthesis also significantly influenced the morphologies. PAz films doped with Cl^–^ showed smooth surfaces; in contrast, Br^–^ resulted in scratchy and rough surfaces. Bulky TOS^–^ anion-induced stress led to a significant overlapping of broken sheet-like structures in the films. With an increased oxidant concentration, PAz films showed substantial growth in the microstructural crowding and linking. The addition of layers displayed a multiplying effect in the properties observed in their respective single-layered films. Pre-peaks in CVs of PAz films exposed the effect of phase segregation and film thickening. Pre-peak splitting was dominant at low scan rates in 3L PAz films synthesized using high concentrations of Cu(II) oxidants. CVs of FeCl_3_ synthesized PAz films exhibited poor capacitance properties. PAz films synthesized using low FeTOS concentration showed pre-peak only at a low scan rate in CVs. PAz films synthesized using high concentrations of Fe(III) oxidants exhibited poor capacitance. In these films, the compact-sheet formations limited slow ion penetration, and restricted conformational changes must have caused this behavior. UV–Vis spectra revealed differences in the oxidized form of PAz films reliant on the oxidant used for synthesis. Enhanced polymer deposition with the concentration of CuCl_2_ and FeTOS and reduced polymer deposition with CuBr_2_ and FeCl_3_ are noticed in the UV–Vis spectra. In addition, a comparison in absorbance due to neutral and oxidized forms of PAz showed that Cu(II) produced films in less oxidized forms than Fe(III) oxidants.

FTIR spectra also revealed that the doping level was high in PAz films synthesized using FeTOS, followed by FeCl_3_, CuCl_2_, and CuBr_2_. This effect is also observed in the intensity of IRAV bands. The radical dication formations dominated the 3L PAz (60 and 120 mM FeTOS), whereas both radical cation and dication formations could be observed for 3L PAz (180 and 240 mM FeTOS). 3L PAz (120 mM CuBr_2_) showed both radical cation and dication formations, whereas radical dication formations dominated 3L PAz (120 mM FeCl_3_) and all CuCl_2_ synthesized films. Linearity in the growth of doped forms in CuCl_2_-synthesized films is justified by IRAV bands and the bands due to radical dication formations. The NIR region of FTIR spectra showed that the increasing FeTOS concentration must have induced the polymerization rate and oxidized form in the resulting PAz films. This may have led to irreversible changes and decomposition due to additional cross-linking at high doping forms. Therefore, despite the highly oxidized form of PAz films, it showed broad absorbance due to radical cation and dication formation in the polymer matrix with the speculated trend of reducing polymer chain length. This elucidation also proves drastic changes observed in UV–Vis spectra, morphologies, and CVs of PAz films synthesized using FeTOS. Similarly, with increasing concentration of CuCl_2_, the NIR region in FTIR spectra suggested reduced polymer chain length even if effective conjugation length may have extended through improved intrachain charge hopping in PAz films.

## Supporting Information Available

AFM images, CVs of AP-VPP PAz films, and list of PAz films prepared and oxidants used in synthesis (PDF)
