# Supplementary material for: Effect of Oxidants on Properties of Electroactive Ultrathin Polyazulene Films Synthesized by Vapor Phase Polymerization at Atmospheric Pressure
Source: Langmuir. 2022 Nov 29;38(49):15165–77. doi: 10.1021/acs.langmuir.2c02215 (PMC9753747; doi:10.1021/acs.langmuir.2c02215)
Supplement: Supplementary file 1 — la2c02215_si_001.pdf [file la2c02215_si_001.pdf]

Effect of oxidant on properties of electroactive ultrathin polyazulene films synthesized by vapor phase polymerization at atmospheric pressure

Rahul Yewale, Pia Damlin, Carita Kvarnström*

Turku University Centre for Materials and Surfaces (MATSURF), Department of Chemistry, University of Turku, FIN-20500 Turku, Finland

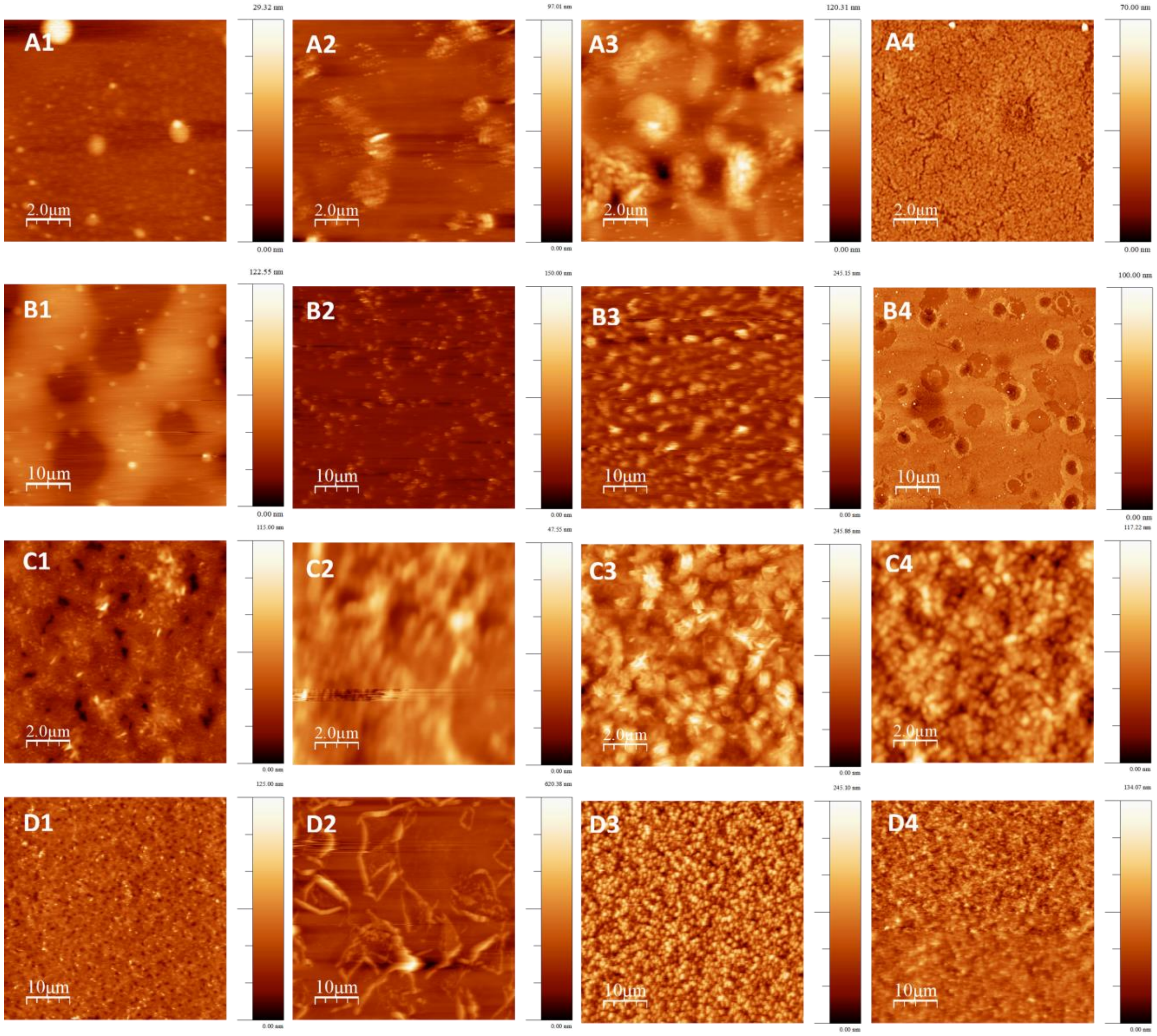

Figure SI 1 AFM images of 1L PAz **A1) and B1)** (120 mM $FeCl_3$), **A2) and B2)** (120 mM $CuBr_2$), **A3) and B3)** PAz (120 mM $CuCl_2$), **A4) and B4)** (120 mM FeTOS), **C1) and D1)** (180 mM $CuCl_2$), **C2) and D2)** (180 mM FeTOS), **C3) and D3)** (240 mM $CuCl_2$), **C4) and D4)** (240 mM FeTOS). (Note: A1 to A4 and C1 to C4 show 10 × 10 μm images. B1 to B4 and D1 to D4 show 50 × 50 μm images).

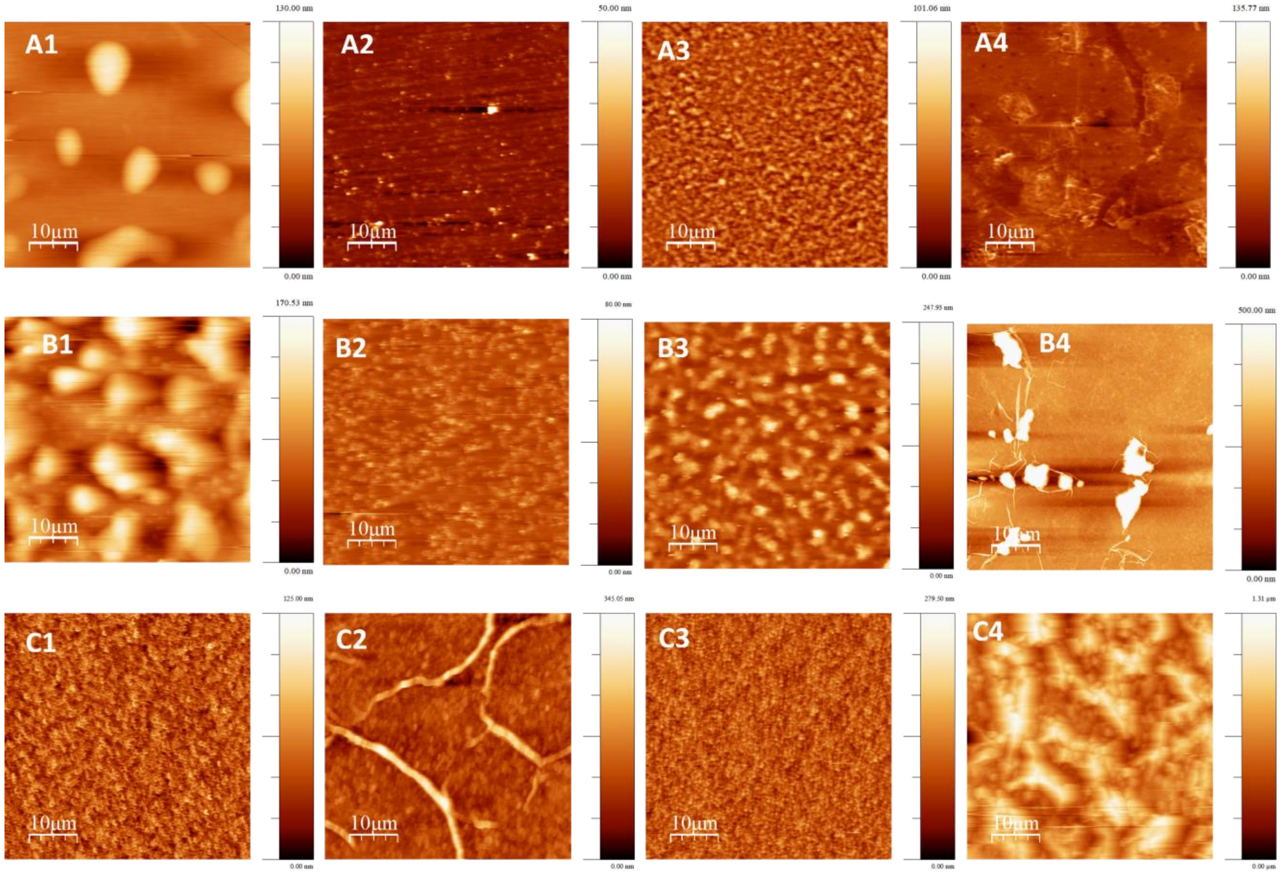

Figure SI 2 AFM images (50 × 50 μm) of 3L PAz **A1)** (60 mM $FeCl_3$), **A2)** (60 mM $CuBr_2$), **A3)** (60 mM $CuCl_2$), **A4)** (60 mM FeTOS), **B1)** 120 mM $FeCl_3$, **B2)** (120 mM $CuBr_2$), **B3)** (120 mM $CuCl_2$), **B4)** (120 mM FeTOS), **C1)** (180 mM $CuCl_2$), **C2)** (180 mM FeTOS), **C3)** (240 mM $CuCl_2$), C**4)** (240 mM FeTOS).

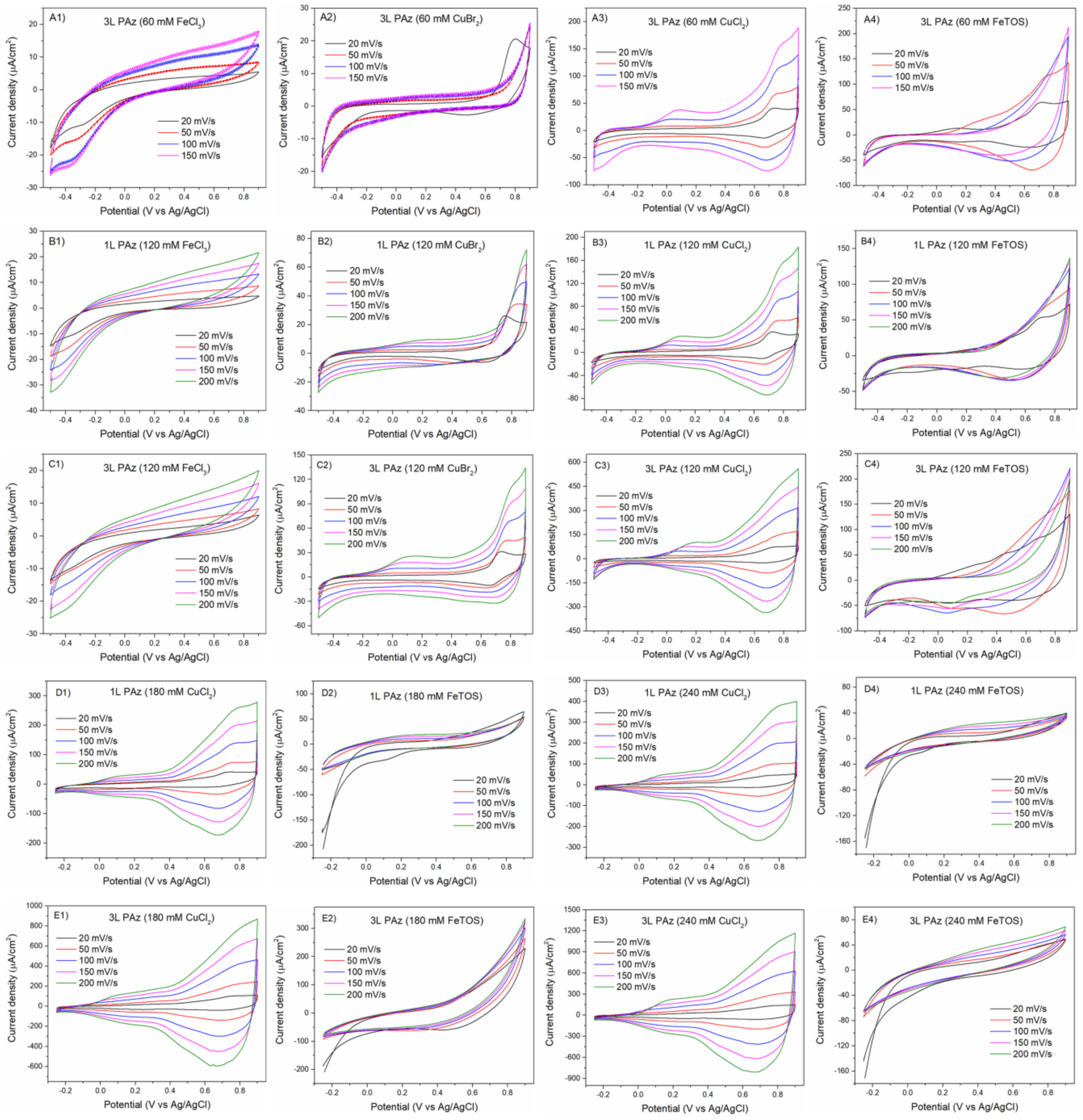

Figure SI 3 CVs of 3L PAz **A1.** (60 mM $FeCl_3$), **A2.** (60 mM $CuBr_2$), **A3.** (60 mM $CuCl_2$), **A4.** (60 mM FeTOS), **C1) (**120 mM $FeCl_3$), **C2)** (120 mM $CuBr_2$), **C3)** (120 mM $CuCl_2$), **C4)** (120 mM FeTOS), **E1)** (180 mM $CuCl_2$), **E2)** (180 mM FeTOS), **E3)** (240 mM $CuCl_2$), and **E4)** (240 mM FeTOS) in 0.1 M TBABF4 in MeCN. CVs of 1L Paz **B1) (**120 mM $FeCl_3$), **B2)** (120 mM $CuBr_2$), **B3)** (120 mM $CuCl_2$), **B4)** (120 mM FeTOS), **D1)** (180 mM $CuCl_2$), **D2)** (180 mM FeTOS), **D3)** (240 mM $CuCl_2$), and **D4)** (240 mM FeTOS) in 0.1 M $TBABF_4$ in MeCN.

Table SI 1 The list of oxidants and their concentrations used in the synthesis, and number of PAz layers in the film and the label used to describe the PAz films

**Oxidant solution (in n-butanol)**	**Number of PAz layers synthesized (L)**	**Label for the PAz film used in the manuscript**
60 mM $CuCl_2$	1	1L PAz (60 mM $CuCl_2$)
	3	3L PAz (60 mM $CuCl_2$)
120 mM $CuCl_2$	1	1L PAz (120 mM $CuCl_2$)
	3	3L PAz (120 mM $CuCl_2$)
180 mM $CuCl_2$	1	1L PAz (180 mM $CuCl_2$)
	3	3L PAz (180 mM $CuCl_2$)
240 mM $CuCl_2$	1	1L PAz (240 mM $CuCl_2$)
	3	3L PAz (240 mM $CuCl_2$)
60 mM FeTOS	1	1L PAz (60 mM FeTOS)
	3	3L PAz (60 mM FeTOS)
120 mM FeTOS	1	1L PAz (120 mM FeTOS)
	3	3L PAz (120 mM FeTOS)
180 mM FeTOS	1	1L PAz (180 mM FeTOS)
	3	3L PAz (180 mM FeTOS)
240 mM FeTOS	1	1L PAz (240 mM FeTOS)
	3	3L PAz (240 mM FeTOS)
60 mM $CuBr_2$	1	1L PAz (60 mM $CuBr_2$)
	3	3L PAz (60 mM $CuBr_2$)
120 mM $CuBr_2$	1	1L PAz (120 mM $CuBr_2$)
	3	3L PAz (120 mM $CuBr_2$)
60 mM $FeCl_3$	1	1L PAz (60 mM $FeCl_3$)
	3	3L PAz (60 mM $FeCl_3$)
120 mM $FeCl_3$	1	1L PAz (120 mM $FeCl_3$)
	3	3L PAz (120 mM $FeCl_3$)
